# Formulation enhanced the stability of Foot-and-mouth virus and prolonged vaccine storage

**DOI:** 10.1186/s12985-022-01928-6

**Published:** 2022-12-03

**Authors:** Jing Li, Yanyan Chang, Shunli Yang, Guangqing Zhou, Yanming Wei

**Affiliations:** 1 College of Veterinary Medicine, Gansu Agricultural University, Lanzhou, China; 2 China Agricultural Vet.Bio.Science and Technology Co, Ltd, Lanzhou, China; 3 State Key Laboratory of Veterinary Etiological Biology, National Foot-and-Mouth Disease Reference Laboratory, Lanzhou Veterinary Research Institute Chinese Academy of Agricultural Sciences, Lanzhou, People’s Republic of China

**Keywords:** FMDV, Formulation, Stability, Vaccine, Shelf life

## Abstract

Foot-and-mouth disease (FMD) is a highly contagious viral disease that affects cloven-hoofed animals. Vaccination is the most effective measure to control FMD. However, FMDV particles are prone to dissociation, leading to insufficient potency of vaccine. Based on this characteristic, a combination of twenty percentage trehalose, 500 mM NaCl and 3 mM CuSO_4_·5H_2_O was developed to increase viral stability. Heating-resistance test showed that FMDV infectivity was maintained when formulated with formulation. Additionally, the half-life of FMDV inactivation was prolonged remarkably. Sequencing analysis demonstrated that viral genome could not be altered in serial passages. Vaccine stability was monitored for up to 1 year at 4 °C, with a higher level of 146S content remained. This study suggested that the formulation could protect FMDV against massive structural breakdown and extend the shelf life of vaccine. Our findings could provide strategy to develop more solutions for the stabilization of viral vaccine.

**Supplementary Information:**

The online version contains supplementary material available at 10.1186/s12985-022-01928-6.

## Background

Foot-and-mouth disease (FMD) is a highly contagious disease that affects cloven-hoofed animals, including pigs, cattle, sheep and many other non-domesticated species [1]. Following FMDV infection, the main clinical signs involve fever, inappetence, lameness and vesicular lesions in snout, mouth, teats and feet [2]. The political and economic impact of this disease include international trade restriction and severe economic losses in many countries [3]. Although FMD has been eradicated in many developed countries, it is still a great challenge to prevent and control in developing countries [4, 5].

Vaccination is a crucial strategy to prevent FMDV infection. Inactivated whole virus vaccine is superior to other vaccines due to the advantage of immunogenicity [6]. However, FMDV particles are prone to dissociation, which often renders the vaccine ineffective.Typically, this requires keeping vaccines refrigerated at all times from production to administration, especially in remote regions of developing countries. The FMDV capsid is composed of 60 copies of VP1, VP2, VP3 and VP4, with five copies of each arranged in pentamers [7]. Previous studies have shown that a cluster of histidine residual located on the interfaces between pentamers and the histidine residues is more likely to be protonated by exposure to elevated temperature, thereby inducing 146S dissociation [8]. Therefore, developing methodologies to enhance FMDV stabilization is a major endeavor.

The development of thermally stable formulations could alleviate the bottlenecks of virus stabilization. For example, Polioviruses exhibited enhanced stability when mixed with 87% deuterium oxide [9]. Cryo-preservative agents were found to protect Live Attenuated Influenza Vaccines (LAIV) protection effectively [10]. Several compounds such as carbohydrates, sugar alcohols and metal ions are commonly used as stabilizers to prevent protein conformational changes, intracellular ice formulation, membrane damage and pH shift during vaccine construction [11, 12]. Among them, trehalose can enhance protein–protein binding by modifying protein hydration properties [13–15]. Metal ions could strengthen the van der Waals attraction forces at the inter-pentameric interface by forming “transition metal ion” [16–18].

Compared with a single stabilizer used alone, a mixture of stabilizers could significantly enhance stabilization of virus. The combinational use of lactalbumin hydrolysate and sucrose has better stabilized effect on attenuated peste des petits ruminants than trehalose alone [19]. Similarly, a camelpox virus was more stable when formulated with a mixture of trehalose-amino acid and divalent cations [20]. These reports suggest the combination of several stabilizers as a good strategy to stabilize viruses. Herein, in this study, we provided a practical formulation composed of trehalose, NaCl and CuSO_4_·5H_2_O, aiming to stabilize FMDV and prolong vaccine shelf life.

## Methods

### Cells and reagent

Baby Hamster Syrian Kidney (BHK-21cells) were cultured in Dulbeccoʼs modified Eagleʼs medium (DMEM) supplemented with 10% FBS. FMDV strains, O/MYA98/BY/2010 and Asia1/JSL/ZK/06 investigated extensively as vaccine strains, were provided by Zhongnongweite biotechnology Co., Ltd. Each strain was propagated in BHK-21 cells at a multiplicity of infection of 0.001 and incubated in 5% CO_2_ at 37 °C. A PrimeScriptTM RT reagent kit containing gDNA Eraser and SYBR Premix Ex TaqTM II (Tli RNaseH Plus) was purchased from TaKaRa (Dalian, China). MTS assay was available from Abcam (Cambridge, UK). The 50% tissue culture infectious dose (TCID_50_) was measured with the Reed and Muench method. Trehalose, CuSO_4_·5H_2_O were purchased from sigma (MO, USA). All other chemicals were analytical grade reagents purchased from Beijing Chemical works (Beijing, China) and all solutions were prepared using Milli-Q grade water (Millipore,USA).

### Screening stabilizers and formulation determination

- Concentrations of Trehalose: 5%, 10%, 20% and 30%
- Concentrations of NaCl: 100 mM, 200 mM, 500 mM and 1 M
- Concentrations of CuSO_4_·5H_2_O: 0.5 mM, 1 mM, 1.5 mM and 3 mM

The thermal stabilization effect of trehalose, NaCl and CuSO_4_·5H_2_O on virus were evaluated by anti-aging test with high performance size exclusion chromatography (HPSEC) method [21]. Briefly, the samples of 100 ul were injected and eluted at 0.6 ml/min with 50 mM phosphate buffer at pH 7.2 containing 100 mM Na_2_SO_4_. The peak area at 259 nm was linearly proportional to 146S concentration with established standard curve. Therefore, 146S content could be calculated according to the peak area. In order to quickly assess the efficiency of stabilizers, formulated virus was incubated at 37 °C for 5 h to accelerate dissociation. Briefly, virus was supplemented with trehalose, NaCl and CuSO_4_·5H_2_O at various concentrations respectively, as detailed in Table 1. Each FMDV was then inactivated with 1 mM binary ethyleneimine (BEI) at 37 °C for 24 h [22]. Subsequently, the formulation was determined as a combination of trehalose, NaCl and CuSO_4_·5H_2_O with optimal concentration screened.

**Table 1:** Stabilization effect of excipients on FMDV 146S at 37 °C for 5 h

Excipients	Concentration (Molarity or % w/v)	Residual 146S (μg/mL)	Degradation rate (%)
Trehalose	(1) 5%	4.8 ± 0.4	76
	(2) 10%	5.2 ± 0.7	74
	(3) 20%	6.5 ± 0.3	67
	(4) 30%	7.1 ± 0.5	64
NaCl	(1) 100 mM	4.1 ± 0.1	79
	(2) 200 mM	5.1 ± 0.1	74
	(3) 500 mM	5.7 ± 0.3	71
	(4) 1 M	4.7 ± 0.3	76
CuSO_4_·5H_2_O	(1) 0.5 mM	5.4 ± 0.1	73
	(2) 1 mM	4.7 ± 0.2	76
	(3) 1.5 mM	5.1 ± 0.2	75
	(4) 3 mM	5.8 ± 0.1	71
No excipient		3.5 ± 0.2	82

### Cytotoxicity assay

MTS assay was performed to measure the cytotoxicity of formulation. In brief, 5 × 10^4^ of BHK-21 cells in 100 μl of complete medium were seeded into each well of a 96-well plate. After incubated at 37 °C for 24 h, the cells were incubated with 100 μl of formulation at various concentrations (1%, 3%, 5%, 10%) for another 72 h. The control cells were treated with DMEM. Then, the supernatants were discarded and 100 μl of DMEM along with 20 μl MTS solution were added to each well for additional 3 h. The cell viability was determined by the percentage of absorbance of the treated cells at 490 nm to that of the control cells.

### Infectivity assay

Infectivity assay were carried out to further confirm the thermal stabilization effect of formulation on virus. After exposed to formulation for 16 h, virus were incubated at room temperature for 3, 5, 10 and 15 h respectively and then used for cells infection. The control virus were incubated for the same periods, but without exposure to the formulation. Briefly, 4 × 10^5^ cells (BHK-21) in 100 μl complete medium were seeded into each well of a 96-well plate. On the following day, the supernatants were discarded, and the cells were washed three times with MEM. Then, the cells were infected with treated virus at serially dilution (eight wells for each concentration) for 1 h. After removing inoculum and adding MEM, the plates were incubated for an additional 24–48 h at 37 °C. Until the cytopathic effect (CPE) was observed, TCID_50_ value was calculated with Reed and Muench method [23]. The supernatants of each well were collected and the viral mRNA were determined by real-time PCR. Briefly, the total RNA from the BHK-21 cells was extracted using TRizol reagent, and 1 µl of RNA was used in reverse transcription reaction using a PrimeScrip™ RT reagent kit containing gDNA Eraser, following the manufacturer’s instructions. The reaction mixture for real-time PCR comprised diluted cDNA (1 µl), 10 µl primers (3DF, 5′-ACTGGGTTTTACAAACCTGTGA-3′; 3DR, 5′-GCGAGTCCTGCCACGGA-3′) and 12.5 µl of SYBR Green Master Mix to a final volume of 25 µl. The amplification conditions were as follows: 95 °C for 30 s, followed by 40 cycles of 95 °C for 5 s, 56 °C for 30 s, and 72 °C for 30 s.

### Inactivation dynamics study

The inactivation rate of virus both in formulated and non-formulated virus were determined after incubation at 25 °C, 37 °C for 36 h and 10 h respectively. Virus were sampled at indicated time points to measure virus titers, referred to TCID_50_ value. According to the log of TCID_50_/ml value over time, the linear curve of inactivation on virus infectivity was established, and the time when the virus lost 50% infectivity was obtained and compared.

### Analysis of genetic stability

To validate if the formulated virus has stable antigen expression throughout multiple passage, the virus genomes of different passage were determined. The conserved VP1 gene was amplified with PCR. Briefly, the formulated virus was continuously passaged for 12 times, then the genomic RNA in every 3 generation were isolated for reverse transcription. The VP1 gene was amplified by PCR in a final volume of 50 μl, containing 1 μl of each primer, 5 μl of PCR buffer, 4 μl of dNTP, 0.25 μl of DNA polymerase, 1 μl of DNA, and 37.75 μl of RNase-free water. The thermocycler conditions were an initial denaturation at 95 °C for 3 min, denaturation with 35 cycles at 95 °C for 50 s.

### Preserving capacity of formulation on FMDV vaccine for long-term storage

Having shown that the formulation could increase the thermostability of infectious FMDV, we speculated that the thermostability of inactivated vaccine would also be enhanced. To facilitate this, both non-formulated and formulated virus were inactivated and then emulsified with adjuvant ISA206 to produce vaccine. The 146S content in vaccine was monitored via HPSEC method during storage at 4 °C for up to 1 year. Samples were collected at indicated time points and then mixed with 1-pentanol in a 10 ml centrifuge tube at a ratio of 9:1. The mixture was shaken fully to break the emulsion. After being placed at 4 °C for 1 h, the upper oil phase was removed and the aqueous phase at the bottom was absorbed slowly for 146S determination.

### Statistical analysis

The means and standard deviations (n = 3) of all values were analyzed with descriptive statistics. The statistical analysis was performed with a t-test, and differences between samples were considered statistically significant at *P* < 0.05.

## Results

### Screen for optimized concentrations of stabilizers to determine formulation

This study was aimed to develop a formulation for FMD virus which has poor thermal stability. Trehalose, NaCl and CuSO_4_·5H_2_O were selected as protectant agents and the optimized concentrations were determined using anti-aging test by detecting residual 146S concentration. The test was performed at 37 °C to quickly screen reasonable concentration. As indicated in Table 1, both formulated and non-formulated FMDV 146S decreased significantly after incubation at 37 °C for 5 h. Whereas, the formulated virus exhibited relatively stable compared to non-formulated one. Among them, twenty trehalose, 500 mM NaCl and 3 mM CuSO4·5H2O was observed to protect virus against dissociation efficiently, with the residual 146S of 6.5 ± 0.3, 5.7 ± 0.3 and 5.8 ± 0.1 μg/ml respectively, thus, the formulated virus was determined to be a combination of twenty percentage trehalose, 500 mM NaCl and 3 mM CuSO_4_·5H_2_O.

**Fig. 1:**
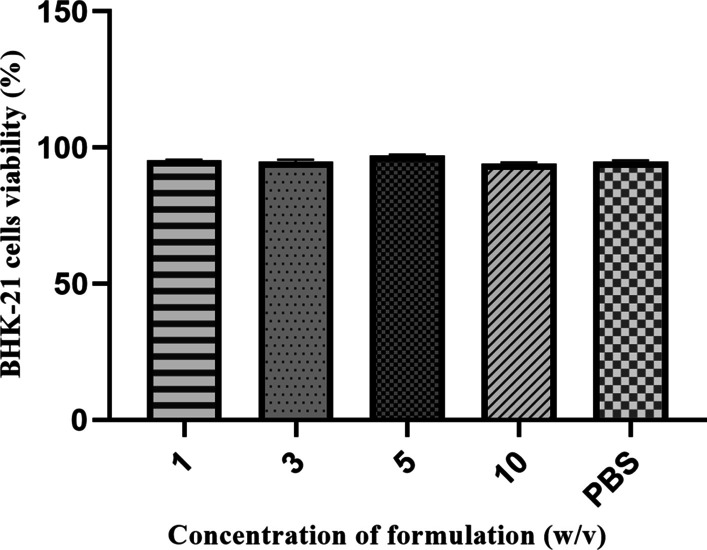
Cytotoxicity of formulation on BHK-21 cells using an MTS assay. BHK-21 cells were treated with different concentration of formulation, and a cell viability was performed via an MTS assay

Cytotoxicity assay were carried out to determine whether the formulation had toxic effects on BHK-21 cells and then attenuated virus infection. As is shown in Fig. 1, the formulation presented no cytotoxicity to cells. The cell viability was 95.3%, 94.8%, 97.2% and 94.2% at formulation concentrations of 1%, 3%, 5% and 10% (w/v) respectively.

### Heating-resistance investigation

**Fig. 2:**
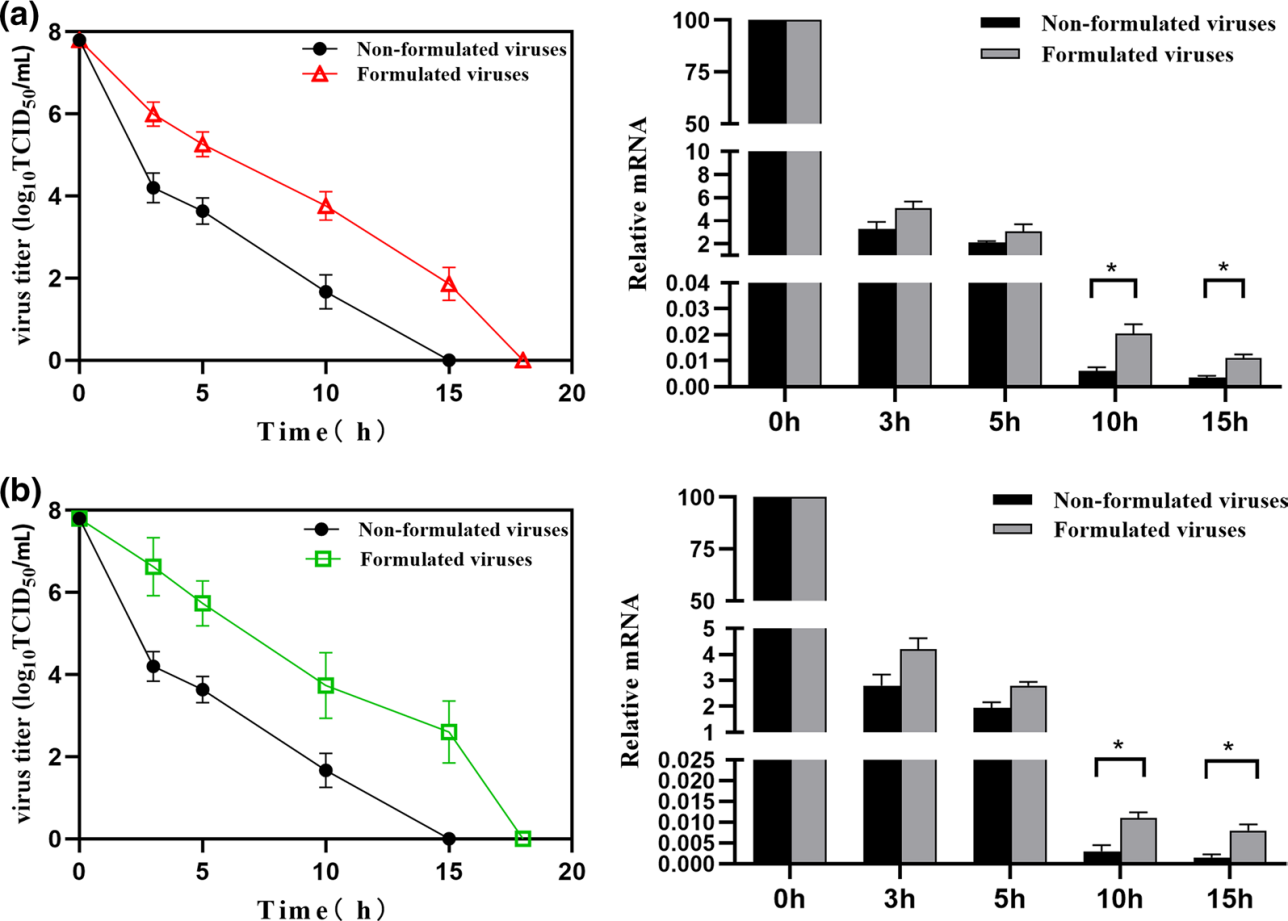
Heating-resistance study. **a** Virus titers and relative mRNA levels determined by pathological cells infected with non-formulated and formulated virus (O/MYA98/BY/2010) after being treated at room temperature for 3, 5, 10 and 15 h. **b** Virus titers and relative mRNA levels determined by pathological cells infected with non-formulated and formulated virus (Asia1/JSL/ZK/06) after being treated at room temperature for 3, 5, 10 and 15 h. Statistically significant differences are indicated by asterisks (*P < 0.05)

To evaluate the thermal stabilization effect of formulation on virus, we firstly investigated the infectivity of the virus by monitoring the TCID_50_ of formulated virus stock at different temperatures. As shown in Fig. 2a, the TCID_50_ values of both formulated and non-formulated virus stock declined in a time-dependent manner during storage at room temperature, but the log_10_TCID_50_/ml values of formulated virus stock (6, 5.3, 3.8 and 1.9) were higher than the non-formulated one (4.2, 3.6, 1.7 and 0) after 3, 5, 10 and 15 h storage. The stable effect of formulation was then verified using real-time PCR analysis. Consistent with the TCID_50_ value, the cell infected with formulated virus had higher relative mRNA level of 4.2%, 2.8%, 0.011% and 0.008%, compared to non-formulated virus (2.8%, 1.95%, 0.003% and 0.002%). Microscopy showed that after treatment at 37 °C for 10 h, the cells inocubated with formulated virus had a higher proportion of infected cells compared to non-formulated virus. Encouragingly, the formulation also show preserving activity for FMDV Asia1/JSL/ZK/06 strain, with log_10_(TCID_50_ /ml) values of 6.6, 5.7, 3.7 and 2.6 (formulated virus) as opposed to 4.2, 3.6, 1.7 and 0 (non-formulated virus). Similarly, the relative mRNA level is higher in formulated virus (5.1%, 3.05%, 0.0205% and 0.011%) than that of non-formulated virus (3.25%, 2.1%, 0.006% and 0.0035%) (Fig. 2b). Taken together, these results suggested that the formulation could stabilize the virus effectively and possess a potential broad-spectrum stabilization activity on other serotype of FMDV viruses.

### Inactivation dynamics study

**Fig. 3:**
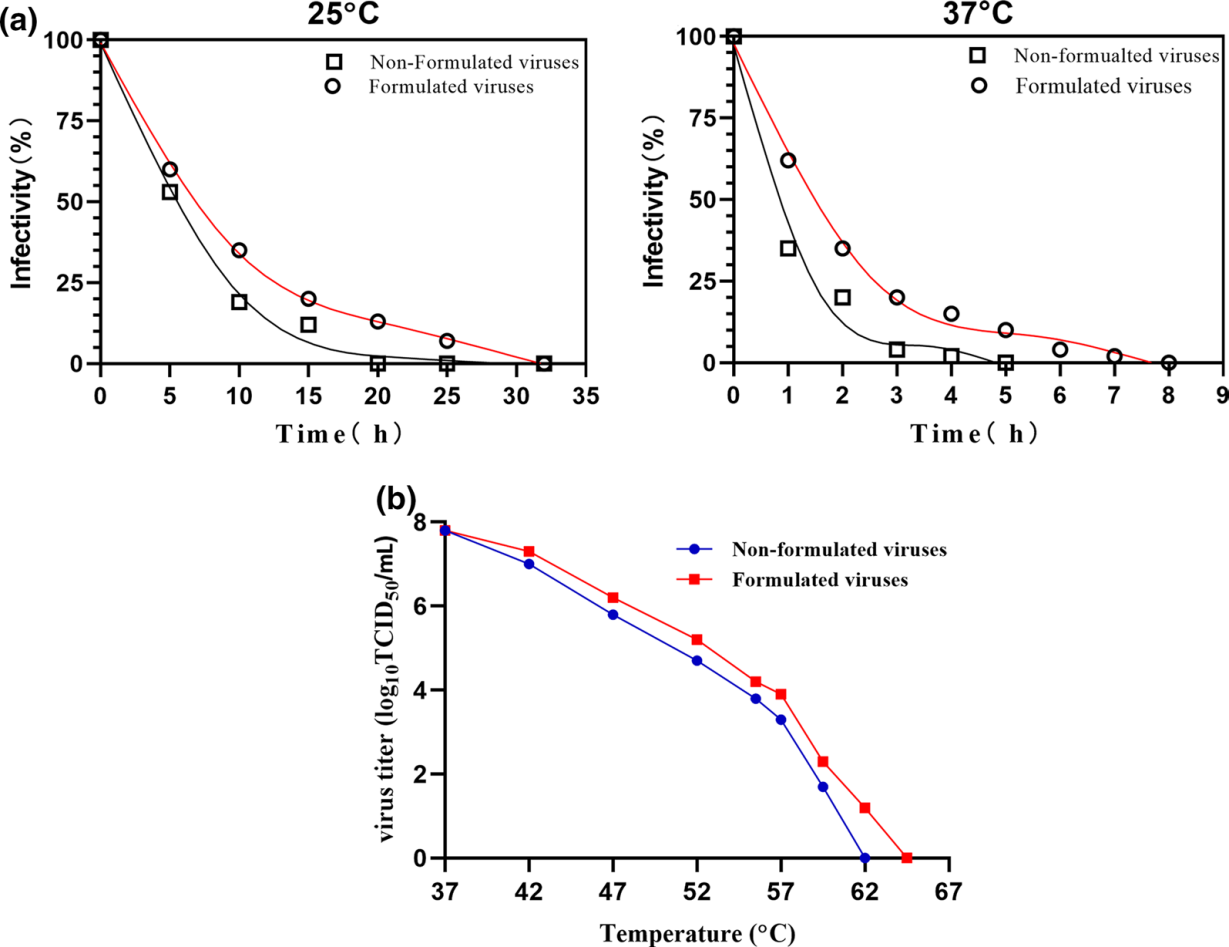
Inactivation kinetics. **a** The percentage of residual infectious virus of non-formulated and formulated viruses were measured after heat treatment at 25 °C, 37 °C for 36 h and 10 h. The samples were collected in regular time points and determined by TCID_50_ value. **b** The temperature required for complete inactivation increased from 62 to 64 °C

The inactivation rate of virus both in formulated and non-formulated were determined after incubation at 25 °C, 37 °C for 36 h and 10 h respectively. Viruses were sampled at regular time points for virus titers detection, referred to TCID_50_ value. As indicated in Fig. 3a, the formulated virus had a lower inactivation rate than the non-formulated virus, and the time against 50% loss in virus infectivity prolonged from 6 to 7 h at 25 °C, 0.9 h to 1.6 h at 37 °C. Also, the temperature required for complete inactivation of formulated virus increased to 64 °C, compared to 62 °C for non-formulated virus (Fig. 3b).

### Identification for viral genetic stability

The stable expression of antigen gene was crucial for vaccine quality. Whether the formulation influence antigenic expression should be fully identified. It showed that the VP1 gene was consistent in continuous 12 passages and the amplification of fragment VP1 yielded 813 bp. In addition, the virus titer in different passages were maintained at relatively steady level (7.0 log10 TCID50/ml) (Additional file 1). These results demonstrated that the main protective antigenic site of FMDV remained stable during virus passage, ensuring the safe and efficiency of vaccine production.

### Evaluation of vaccine stability on storage

**Fig. 4:**
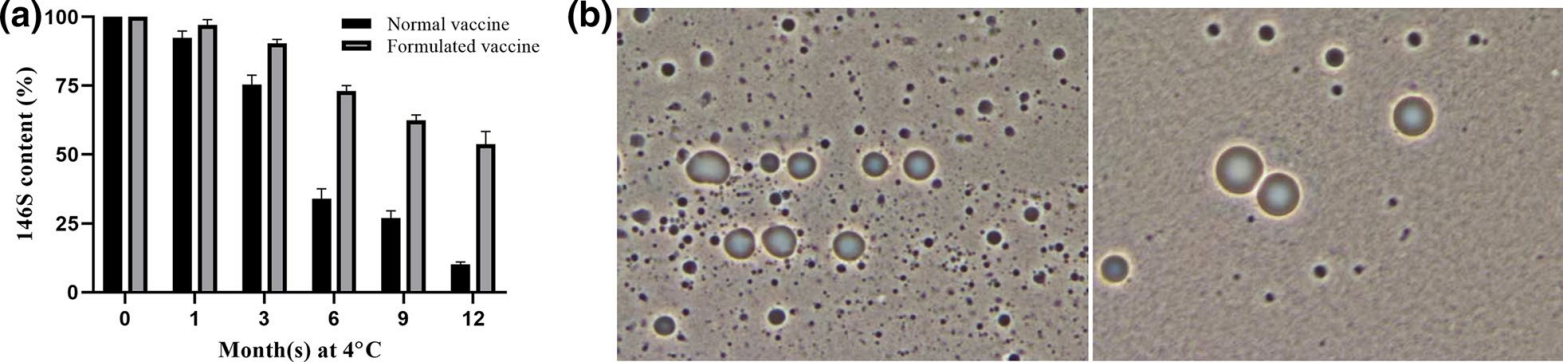
Vaccine stability study. **a** The shelf life of normal and formulated vaccine stored at 4 °C. **b** Micrographs of normal (b-1) and formulated vaccine (b-2) at 1000 ×

Reducing dependence on cold storage and improving the stability of vaccine are two objectives of developing vaccine formulation. In order to further evaluate the efficiency of formulation on vaccine, we investigated the shelf life of vaccine stored at 4 °C. The 146S concentration indicates intact FMDV particles and therefore is a crucial parameter to assess vaccine quality. We applied HPSEC to monitor the 146S concentration considering its high accuracy, high sensitivity and requiring less time. As shown in Fig. 4a, the formulated vaccine exhibited excellent stability, half of the 146S remained even stored at 4 °C for 12 months, indicative of effective protection. However, the normal vaccine (prepared with inactivated virus for no formulation) resulted in a time-dependent loss in 146S concentration, only 10% remained. Furthermore, the dosage form of the formulated vaccine is W/O/W, rarely show any morphological abnormality (Fig. 4b). Collectively, these observations suggested the stabilizing effect of formulation on vaccine, allowing for its effective stockpiling.

## Discussion

Since FMDV is highly unstable and can easily dissociate into less immunogenic 12S particles under the general vaccine production processes, preserving the virus is challenging. Several classes of excipients have been proven to be effective for virus stabilization. Moreover, individual stabilizing capacity of each stabilizer can work synergistically for Marek' [24], Classical swine fever [25] and Newcastle Disease [26]. Thus, considering the efficacy, safety and stability, it might also be employed in FMDV.

Previous studies have shown that trehalose, NaCl could act as filler, electric attraction inducer to strengthen the interaction between virus interpentameric. Cu^2+^, linked between adjacent histidine and other amino acid at the inter-pentameric interface of the capsids could enhance both the thermostability and acid -resistant stability of capsids [27]. So these agents were chose to be components of formulation, and the optimal concentration of these components were determined by anti-aging test and HPSEC. Although thirty percentage trehalose exhibited better stabilizing effect than twenty percentage trehalose, it was so viscous that could burden filtration process, therefore we finally used twenty percentage trehalose in the following test. Compared with other tested group, the low degradation of virus was observed in 500 mM NaCl and 3 mM CuSO_4_·5H_2_O, with residual 146S of 5.7 and 5.8 μg/ml respectively when treated at 37 °C for 5 h. Collectively, twenty percentage trehalose, 500 mM NaCl and 3 mM CuSO_4_·5H_2_O were determined as compositions for the formulation.

MTS assay results revealed that the formulation have no cytotoxcity effect on BHK-21 cells when treated with 1%, 3%, 5% and 10% (w/v) formulation, with cell viability of 95.3%, 94.8%, 97.2% and 94.2%. In addition, TCID_50_ value and real-time PCR findings showed that the formulation could significantly stabilize not only serotype O, but also serotype Asia1 FMDV strain. Among the seven serotypes of FMDV, serotypes O and SAT are, in particular, more unstable. Similarly, in this study we also found that the TCID_50_ value and relative mRNA level in serotype O are lower than that of serotype Asia1 FMDV. Collectively, these results indicated that the formulation could be a potential broad-spectrum stabilizer for FMDV. According to inactivation dynamic study, the time inducing 50% loss in activity of formulated viruses prolonged, meanwhile, the temperature required for complete inactivation was also increased. To investigate the genetic stability of formulated viruses in continuously passage, VP1 in F3, F6, F9 and F12 was identified to be consistent and the virus titer retained approximately 7.0 log_10_ TCID_50_/ml, almost be equal to that of non-formulated virus.

Although the formulation was identified to protect virus against dissociation, it was uncertain about the situation on vaccine. In this study, we monitored the changes of 146S content in vaccine via real-time analyses and the result showed that half of 146S was remained after stored at 4 °C for up to 1 year. Overall, in the present study, we validated that the adoption of formulation for FMDV preservation is valuable for enhancing virus stabilization and meanwhile prolonging vaccine shelf life.

Purification is a crucial production process determining vaccine quality. Owing to the advantage of scalability and high selectivity, ion exchange chromatography is an alternative strategy for FMDV antigen purification. However, Shanqing Liang et al. reported that the interaction between inactivated FMDV and ion media could cause capsid denaturation and then induced dissociation, hardly obtaining high recovery and stable native structural antigen[28]. In this study, we have identified that the formulation was involved in stabilizing effect on viral structural integrity, thus, further studies should investigate whether the formulation could enhance antigen stability in the production process of purification, providing technical support for manufacturing facilities.

## Conclusion

In summary, a formulation for FMDVstability was screened and determined with HPSEC method. The formulation could maintain viral integrity effectively even with elevated temperature and has no influence on genetic stability during viral passages. Monitoring of vaccine stability on long-term storage was further confirmed its stable effect on FMD vaccine. This research is a step forward in vaccine preservation protocols and could be a potential application in vaccine production regimens for FMD prevention.

## Supplementary Information

**Additional file 1**. Genetic stability of formulated FMDV. (a) Amplification of VP1 genes from different viral passages. Lane M: 2000bpDNA Maker; Lane1: F3; Lane2: F6; Lane3: F9; Lane4: F12. (b) Comparison of virus titer between non-formulated and formulated viruses during 12 passage cycles.

## Data Availability

No applicable.
