# Supplementary material for: Formulation enhanced the stability of Foot-and-mouth virus and prolonged vaccine storage
Source: Virol J. 2022 Dec 3;19:207. doi: 10.1186/s12985-022-01928-6 (PMC9719126; doi:10.1186/s12985-022-01928-6)

**Genetic stability of formulated FMDV.** (a) Amplification of VP1 genes from different viral passages. Lane M: 2000bpDNA Maker; Lane1: F3; Lane2: F6; Lane3: F9; Lane4: F12. (b) Comparison of virus titer between non-formulated and formulated viruses during 12 passage cycles.

bp

750050

M

F3

F6

F9

F12


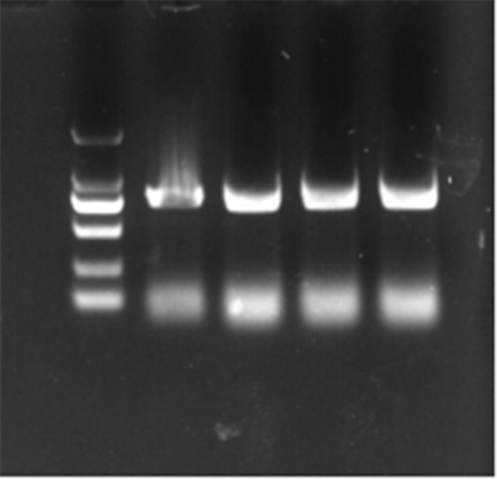


1000

2000

250

100

500


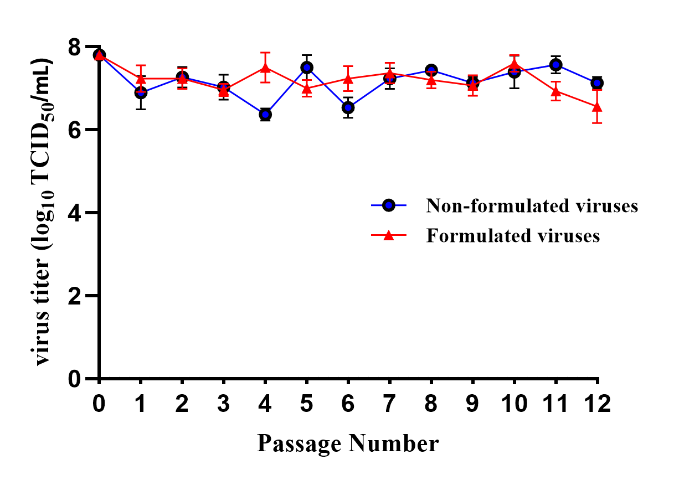

Supplement: Supplementary file 1 — Additional file 1. Genetic stability of formulated FMDV. (a) Amplification of VP1 genes from different viral passages. Lane M: 2000bpDNA Maker; Lane1: F3; Lane2: F6; Lane3: F9; Lane4: F12. (b) Comparison of virus titer between non-formulated and formulated viruses during 12 passage cycles. [file 12985_2022_1928_MOESM1_ESM.docx]
